# Cancer Screening in Hard-to-Reach Populations

**DOI:** 10.3934/publichealth.2017.4.399

**Published:** 2017-08-01

**Authors:** Paul D. Terry

**Affiliations:** Department of Medicine, The University of Tennessee Graduate School of Medicine, Knoxville, Tennessee, USA

We are pleased to present this special issue on cancer screening. While guidelines for cancer screening are continually refined, the early detection of treatable cancer is one of the most important public health priorities of our time. Cancer screening saves lives. Some populations have particularly low participation in screening, and consequently suffer higher rates of cancer morbidity and mortality. That is unacceptable, and it is why we produced this special issue. Although the scientific methods and populations described in the various studies presented within this issue are distinct, they converge in their pursuit of one goal: to reduce cancer morbidity and mortality in hard-to-reach populations through screening.

Karent Zorogastua and colleagues [1] address the underuse of breast and cervical cancer screening among African American and African-born Muslim women in New York City. Using mixed methods, including focus groups, the authors shed new light on religious and health beliefs and attitudes that influence screening behavior in their study population. The study results will be useful for researchers seeking strategies to increase breast and cervical cancer screening.

Dr. Eleni Tolma and colleagues [2] describe the design of a multi-component intervention, the Native Women's Health Project, among American Indian/Alaska Native women living in Oklahoma. The authors used clinical and community-based approaches to discern factors related to breast cancer screening behavior, and with that information partnered with members of the community to promote cancer screening. This article highlights some of the complexities of conducting research and promoting cancer screening in hard-to-reach populations.

Dr. Yan Lin and colleagues [3] used survey methods to examine barriers to breast, cervical, and colorectal cancer screening among female American Indians in South Dakota, a state with over 70,000 residents of native American ancestry. The authors note the high cancer burden experienced by members of this group, as well as several factors that make outreach difficult, including the remoteness of Native American land, high household mobility, high proportion of households without phone or internet access, and their tendency to distrust “outsiders”. The authors explore how geography, study participants' knowledge about cancer screening, their access to primary care, and their socioeconomic status, may influence cancer screening behavior. The study findings and conclusions are of universal interest to those seeking to develop cancer screening programs in hard-to-reach populations.

Caroline B. T. Makura and colleagues [4] used district-level choropleth maps (different colors on a geographical map representing different levels of a variable) to show variations in pap smear coverage, pap smear quality, and the burden of high-grade cytological abnormalities across South Africa, with consideration of the HIV-positive sub-population. This article shows the vital importance of descriptive epidemiology in areas of the world where basic studies of cancer screening may be lacking.

From the School of Nursing and Midwifery at Western Sydney University, Dr. Cannas Kwok [5] notes the low participation in cancer screening of several immigrant groups in Australia. Immigrants from underdeveloped countries tend to be relatively unwilling to participating in research, including research seeking to improve disease prevention and treatment in those same individuals. It seems a “vicious cycle” of non-participation, which tragically ends with a poor quality of life in the cancer survivorship state. To break this cycle, Dr. Kwok notes the importance of establishing partnerships with ethnic community organizations (ECOs) through engagement, ongoing support, adequate resources, and open acknowledgment of the vital roles ECOs and participants play in research. Dr. Kwok's distillation of her successful experience working with immigrants in cancer research provides thoughtful reading on this important topic.

Additional related articles may appear in this issue as time moves forward. Meanwhile, we are proud to have worked with these authors to bring you a synergistically informative group of articles in this special issue on cancer screening in hard-to-reach populations.
